# Evolving Aerobic Bacterial Skin Flora and Healthcare-Associated Infections Among Intensive Care Unit Patients at a Tertiary Care Hospital: A Therapeutic Concern

**DOI:** 10.7759/cureus.102408

**Published:** 2026-01-27

**Authors:** Utsav Gupta, Garima Kapoor, Ravpreet Kaur, Naveen Patbamniya, Deepak Nayak, Harsh Darshan, Ashish Tiwari

**Affiliations:** 1 General Surgery, Gandhi Medical College, Bhopal, IND; 2 Microbiology, Gandhi Medical College, Bhopal, IND; 3 Community Medicine, Institute of Medical Sciences, Banaras Hindu University, Varanasi, IND; 4 General Surgery, Teerthanker Mahaveer Medical College and Research Centre, Moradabad, IND

**Keywords:** aerobic bacteria, hai, hospital-acquired infections, icu, nosocomial infections, skin flora, who priority pathogens

## Abstract

This study aimed to assess the evolution of aerobic bacterial flora among ICU patients and its association with healthcare-associated infections in a tertiary care hospital. A total of 52 patients were selected from the medical, surgical, and burn and plastic surgery ICUs. A total of 764 skin swabs were collected from four sites, namely the fingertips and webs, dorsum of the hand, axilla, and anterior nares on days 1, 2, 3, 4, and 7.

Of the total swabs collected, 484 (63.4%) were identified to have WHO priority pathogens and potentially pathogenic bacteria. A total of 650 isolated microorganisms were isolated from the 484 swabs, comprising both WHO priority pathogens and potentially pathogenic bacteria. Fingertips and webs were found to have the highest pathogenic burden of these 650 microorganisms (27.7%, n=180), with WHO priority pathogens comprising 20.5% (n=37) and extended-spectrum beta-lactamase (ESBL)-producing carbapenem-resistant (CRP-R) Enterobacteriaceae being the most prevalent (9.4%, n=17). Other potentially pathogenic bacteria accounted for 79.4% (n=143), led by vancomycin-sensitive methicillin-resistant *Staphylococcus aureus* (MRSA) (26.7%, n=48).

On day 1 of hospitalization, the total culture count was 208, and on day 7, it was 100. The critical WHO priority pathogen colonization on day 1 increased significantly from 6.7% (n=14) to 27% (n=27) by day 7 (p<0.05). The burn and plastic surgery ICU (total n of swabs collected=152) had the highest microbial load, with *Pseudomonas aeruginosa* (30.2%, n=47) and carbapenem-resistant strains being predominant. This study reveals that colonization of multidrug-resistant organisms increases with prolonged hospitalization in ICUs, highlighting the need for strict infection control protocols. Regular monitoring and effective hygiene practices can reduce the spread of hospital-acquired infections (HAIs) and improve patient outcomes.

## Introduction

Skin, the largest organ of the human body, serves as the primary barrier against pathogens invading through the environment [1]. It hosts diverse microbiota, which influences the host's anatomy, physiology, and susceptibility to infections [1]. However, the skin flora can also serve as a source of infection, particularly in hospitalized patients [2]. With the rise of multidrug-resistant (MDR) organisms and the inappropriate use of antibiotics, hospital-associated microflora is rapidly evolving, contributing to the transmission of infections among both patients and healthcare workers [3,4].

Hospital-associated infections (HAIs) are infections acquired in the hospital by a patient who was admitted for a reason other than that infection and not present or incubating at the time of admission [5]. Global prevalence studies indicate that 8.7% of hospitalized patients develop HAIs, with higher rates observed in the Eastern Mediterranean (11.8%) and Southeast Asia (10%) regions, particularly in ICUs and among neonates [5,6]. In low- and middle-income countries, the prevalence is as high as 15.5%, as per WHO reports [6].

This study was designed to assess the changes in skin flora of ICU patients who are at higher risk for developing HAIs by screening common sites of bacterial colonization, including the fingertips and webs, dorsum of the hand, axilla, and anterior nares. Previous studies have not comprehensively evaluated bacterial flora across these sites nor investigated variations in flora over time during hospital stays. Therefore, the study aims to categorize the isolated flora into WHO priority pathogen groups 1, 2, and 3 and other bacteria with pathogenic potential, including MDR organisms such as extended-spectrum-beta-lactamase (ESBL) producers, carbapenem-resistant Enterobacteriaceae (CRE), and methicillin-resistant *Staphylococcus aureus *(MRSA), and examine changes in flora with prolonged hospital stays. Additionally, the study evaluates the association of skin flora with nosocomial infections in medical, surgical, and burn and plastic surgery ICU patients. Surveillance of bacterial flora may offer insights into the timing of effective interventions to prevent HAIs and reduce associated morbidity.

## Materials and methods

This longitudinal study was conducted at a tertiary care hospital associated with Gandhi Medical College, Bhopal, India, over a period of three months (May to August 2024) with approval from the Institutional Ethics Committee (approval no. 2107374/MC/IEC/2024). All consenting patients admitted to the medical, surgical, and burn and plastic surgery ICUs during the study period and who were at high risk for nosocomial infections (due to catheterization, mechanical ventilation, etc.) were included. Patients with a history of antibiotic intake or hospital admission in the past three months, those on home infusion therapy or chronic dialysis, and patients with trauma at sample collection sites were excluded (due to likely contamination).

A total of 52 patients (n=23 from medical, n=18 from surgical, and n=11 from burn and plastic surgery ICUs) were enrolled in the study. Skin swabs were collected from four sites, i.e., fingertips and webs (site A), dorsum of hand (site B), axilla (site C), and anterior nares (site D). Samples were taken on day 1 (at the time of admission), day 2 (at 24 hours), day 3 (at 48 hours), day 4 (at 72 hours), and day 7 (to assess change in aerobic bacterial flora) during hospitalization. If patients were transferred from the ICU to the wards, they were followed for sample collection until day 7.

While the target was to collect 1040 swabs (52 patients × 20 swabs from each patient), only a total of 764 swabs were collected (n=304 from medical, n=152 from burn and plastic surgery, and n=308 from the surgical ICUs). This was due to patients leaving against medical advice (LAMA), deaths, or other complications such as local injury or inflammation at the sample collection sites. In addition, appropriate specimens (n=15) for nosocomial infections were collected from 11 patients (n=11 from burn and plastic surgery, n=3 from surgery, and n=1 from medical ICU), and relevant clinical signs were collected via a data collection form (see Appendix A). Samples were collected aseptically using sterile swabs moistened with saline and processed using conventional microbiological techniques, where bacteria were identified through aerobic cultures on blood and MacConkey agar.

Antimicrobial susceptibility was determined via the Kirby-Bauer disk diffusion method, categorized by Clinical Laboratory Standard Institute (CLSI) 2024 guidelines [7]. For the screening of MRSA, a 30 μg cefoxitin disk was used on Mueller-Hinton agar (MHA). A zone diameter of ≤21 mm was interpreted as positive for MRSA, while a diameter of ≥22 mm was considered negative, because cefoxitin is used as a surrogate marker for detecting mecA-mediated oxacillin resistance. For ESBL screening, ceftazidime (30 μg) and ceftazidime-clavulanate (30/10 μg), as well as cefotaxime (30 μg) and cefotaxime-clavulanate (30/10 μg), were used on MHA. An increase in zone diameter of ≥5 mm for the antimicrobial agent tested in combination with clavulanate compared to when tested alone was considered indicative of ESBL production (e.g., ceftazidime zone = 16 mm; ceftazidime-clavulanate zone = 21 mm). For the screening of vancomycin-resistant *S. aureus* (VRSA), Vancomycin Ezy MIC™ strips (HiMedia Laboratories Pvt. Ltd., Mumbai, MH, IND) were used to determine the minimum inhibitory concentration (MIC). The MIC values of ≤2 µg/ml were classified as vancomycin-sensitive, 4 µg/ml to 8 µg/ml as vancomycin-intermediate, and ≥16 µg/ml as vancomycin-resistant (V-R). The bacteria isolated were categorized according to the WHO priority pathogen list for research and development of new antibiotics into priority groups 1, 2, and 3 [8], and ESBL producers, CRE, MRSA, and potentially clinically insignificant bacteria.

Data were entered in Microsoft Excel 2010 (Microsoft Corp., Redmond, WA, USA) and analyzed using the SPSS Statistics trial version (IBM Corp., Armonk, NY, USA). Frequencies with percentages for categorical variables were calculated to summarize patient demographics and pathogen colonization rates. The chi-square test for trend was used to determine statistically significant differences. A p-value of <0.05 was considered statistically significant.

## Results

A total of 1250 organisms were isolated from the 764 swabs collected. Of these, 484 swabs (63.4%) were positive for 650 microorganisms that were WHO priority pathogens and other potentially pathogenic bacteria; 280 swabs (36.6%) yielded 600 non-pathogenic bacteria (*Micrococcus spp.*, coagulase-negative staphylococci, diphtheroids, and aerobic spore bearers). Since the focus of our study is to isolate and categorize pathogenic bacteria and assess their colonization and pathogenicity over time, we excluded all non-pathogenic bacteria from the analysis (Table 1).

**Table 1:** Distribution of pathogens isolated from swabs collected from all four sites of study subjects admitted to different ICUs V-R: Vancomycin-resistant, V-S: Vancomycin-susceptible, VRSA: Vancomycin-resistant *S. aureus*, ESBL: Extended-spectrum beta-lactamase, CRP-S: Carbapenem-susceptible, CRP-R: Carbapenem-resistant, MRSA: Methicillin-resistant *S. aureus*, MSSA: Methicillin-sensitive *S. aureus*

Microorganism	Total n=650	Percentage of 650 isolated microorganisms	Percentage of 484 positive cultures	ICUs		
				Burn and plastic surgery	Surgery	Medicine
WHO *Acinetobacter*	19	2.9%	3.9%	3	6	10
WHO *Pseudomonas*	42	6.5%	8.7%	35	3	4
WHO Enterobacteriaceae	62	9.5%	12.8%	14	19	29
WHO *Enterococcus* V-R	4	0.6%	0.8%	2	0	2
WHO VRSA	15	2.3%	3.1%	0	10	5
WHO medium category	0	0	0	0	0	0
Pathogenic *Acinetobacter*	17	2.6%	3.5%	1	6	10
Pathogenic *Pseudomonas*	19	2.9%	3.9%	11	1	7
Pathogenic Enterobacteriaceae ESBL; carbapenem-susceptible (CRP-S)	18	2.8%	3.7%	7	5	6
Pathogenic Enterobacteriaceae non-ESBL; CRP-S	94	14.5%	19.4%	26	38	30
Pathogenic Enterobacteriaceae non-ESBL; CRP-R	107	16.5%	22.1%	65	15	27
Pathogenic *Enterococcus* vancomycin-susceptible (V-S)	5	0.8%	1.0%	2	0	3
Pathogenic MRSA	195	30.0%	40.3%	38	93	64
Pathogenic methicillin-sensitive *S. aureus* (MSSA)	53	8.2%	11.0%	5	24	24

**Figure 1:**
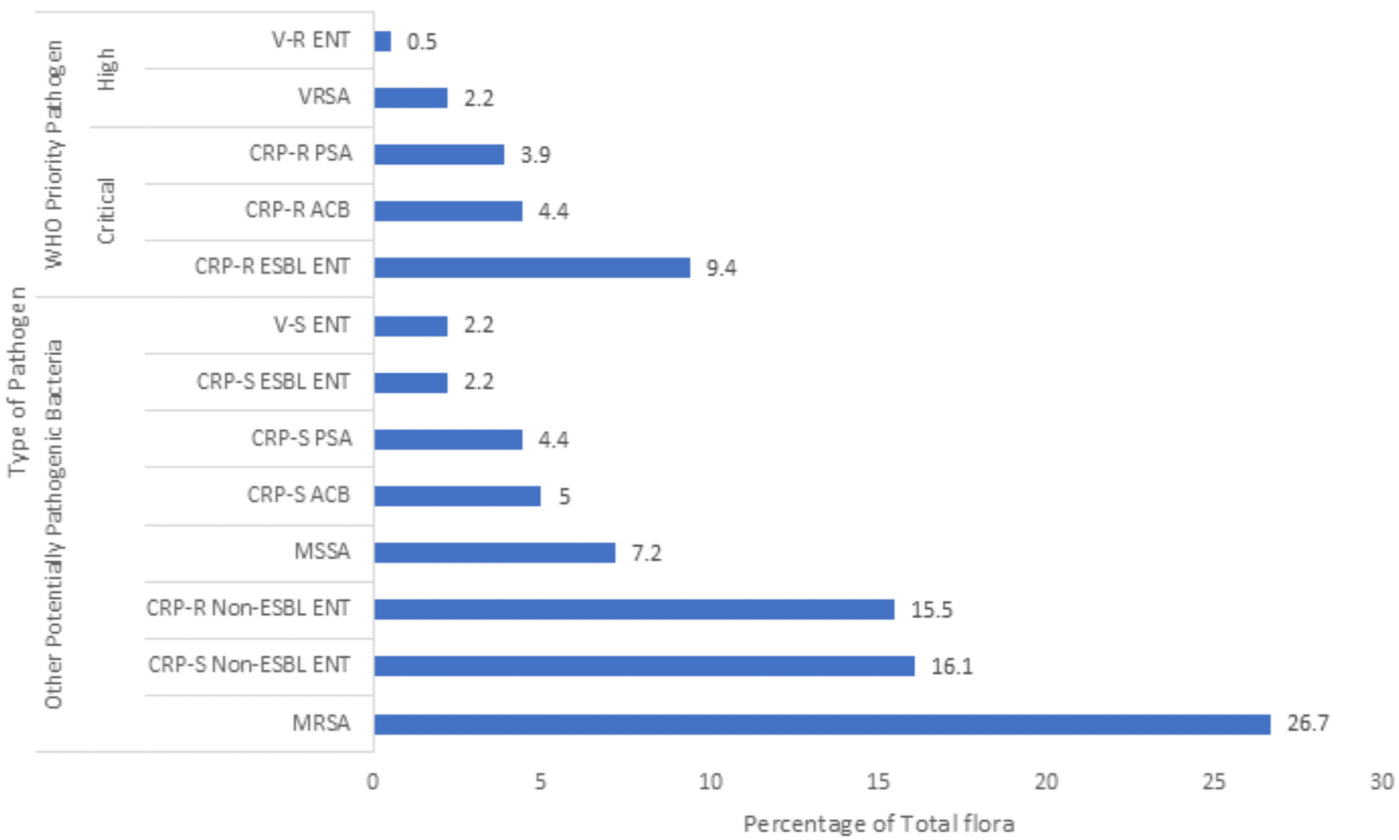
Bacterial flora isolated from site A MRSA: Methicillin-resistant *S. aureus*, MSSA: Methicillin-sensitive *S. aureus*, ENT: Enterobacteriaceae, PSA: *Pseudomonas aeruginosa*, ACB: *Acinetobacter baumannii*, CRP-S: Carbapenem-susceptible, CRP-R: Carbapenem-resistant, ESBL: Extended-spectrum-beta-lactamase, V-S: Vancomycin-susceptible, V-R: Vancomycin-resistant, ENT: Enterococci, VRSA: Vancomycin-resistant *S. aureus; *Site A: Fingertips and webs

**Figure 2:**
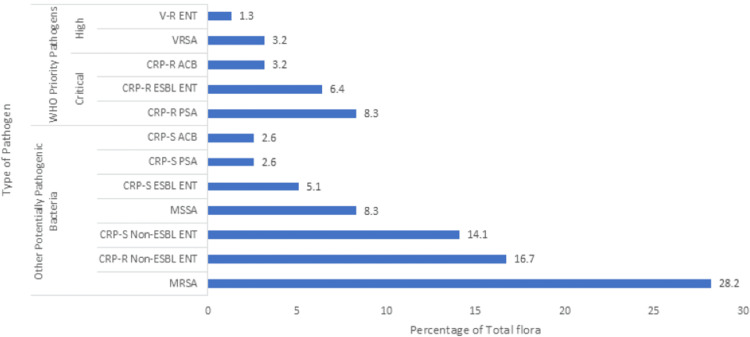
Bacterial flora isolated from site B MRSA: Methicillin-resistant *S. aureus*, MSSA: Methicillin-sensitive *S. aureus*, ENT: Enterobacteriaceae, PSA: *P. aeruginosa*, ACB: *A. baumannii*, CRP-S: Carbapenem-susceptible, CRP-R: Carbapenem-resistant, ESBL: Extended-spectrum-beta-lactamase, V-S: Vancomycin-susceptible, V-R: Vancomycin-resistant, ENT: Enterococci, VRSA: Vancomycin-resistant *S. aureus*; Site B: Dorsum of hand

**Figure 3:**
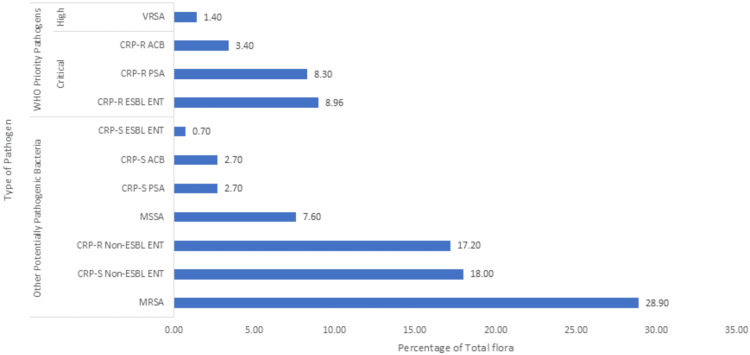
Bacterial flora isolated from site C MRSA: Methicillin-resistant *S. aureus*, MSSA: Methicillin-sensitive *S. aureus*, ENT: Enterobacteriaceae, PSA: *P. aeruginosa*, ACB: *A. baumannii*, CRP-S: Carbapenem-susceptible, CRP-R: Carbapenem-resistant, ESBL: Extended-spectrum-beta-lactamase, V-S: Vancomycin-susceptible, V-R: Vancomycin-resistant, ENT: Enterococci, VRSA: Vancomycin-resistant *S. aureus*; Site C: Axilla

**Figure 4:**
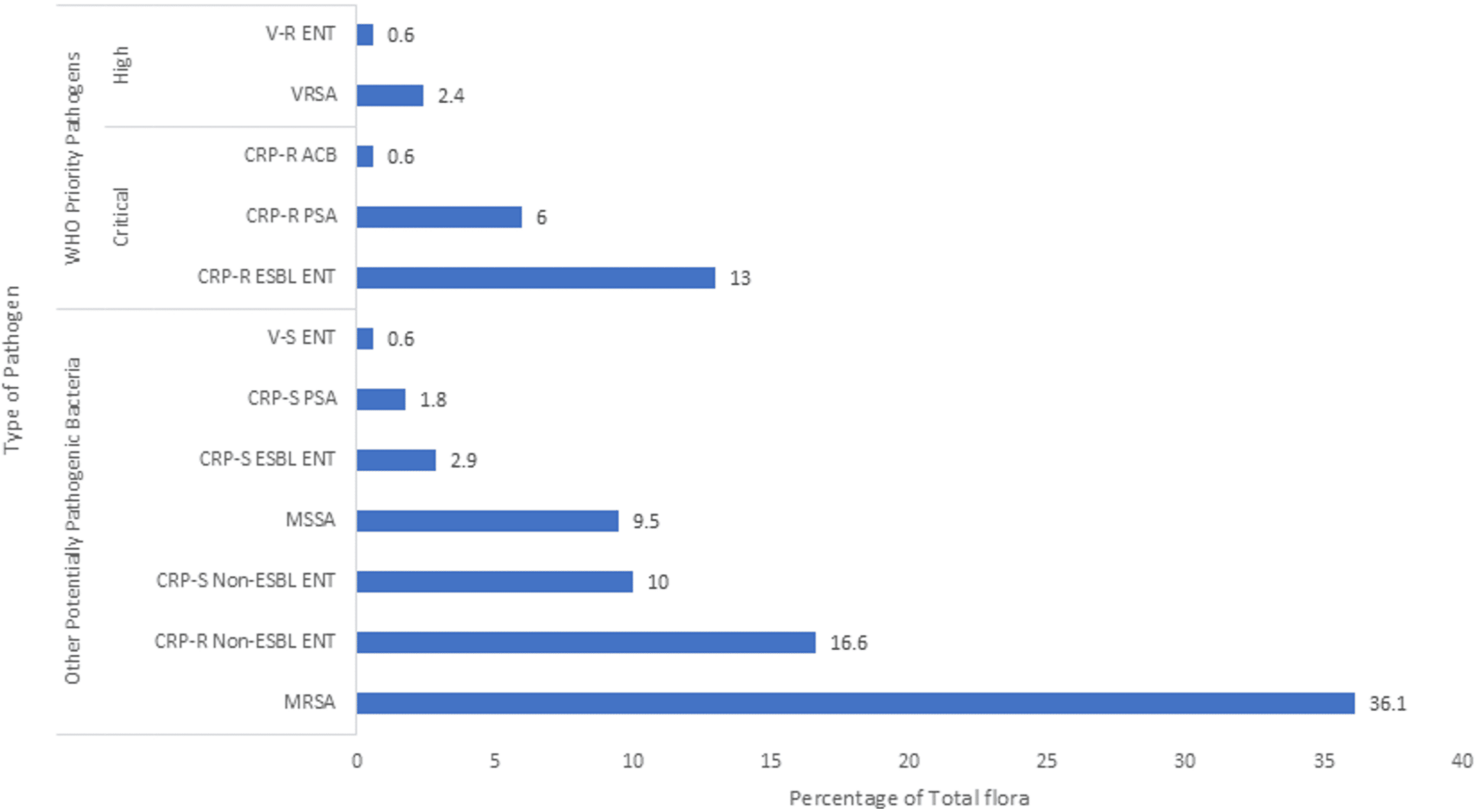
Bacterial flora isolated from site D MRSA: Methicillin-resistant *S. aureus*, MSSA: Methicillin-sensitive *S. aureus*, ENT: Enterobacteriaceae, PSA: *P. aeruginosa*, ACB: *A. baumannii*, CRP-S: Carbapenem-susceptible, CRP-R: Carbapenem-resistant, ESBL: Extended-spectrum-beta-lactamase, V-S: Vancomycin-susceptible, V-R: Vancomycin-resistant, ENT: Enterococci, VRSA: Vancomycin-resistant *S. aureus*; Site D: Anterior nares

The overall burden of pathogenic flora, including WHO priority pathogens and other potentially pathogenic bacteria (total n=650), varied across anatomical sites in the patient population (Figures 1-4). On site A, the total burden of isolated microorganisms was 27.7% (n=180). Of these 180, WHO priority pathogens accounted for 20.5% (n=37), while other potentially pathogenic bacteria made up 79.4% (n=143). At site B, the total burden was slightly lower at 24.0% (n=156), with WHO priority pathogens representing 22.4% (n=35) and other bacteria comprising 77.6% (n=121). Site C showed a total burden of 22.3% (n=145), where WHO priority pathogens constituted 22.0% (n=32) and other potentially pathogenic bacteria accounted for 77.9% (n=113). Finally, site D had a total burden of 26.0% (n=169), with WHO priority pathogens comprising 22.5% (n=38) and other bacteria making up 77.5% (n=131).

**Figure 5:**
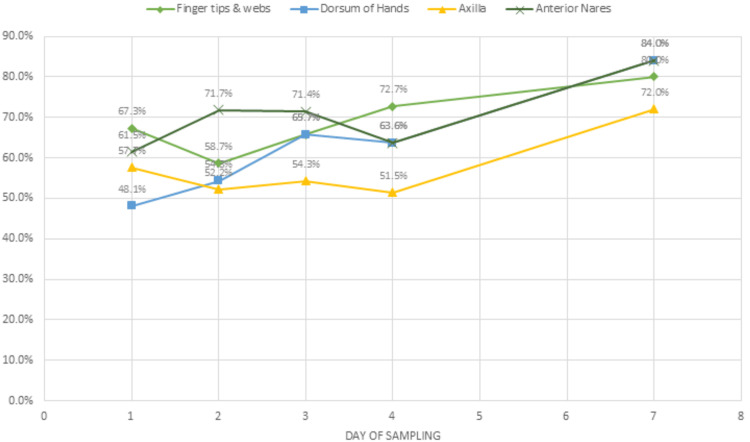
Site and day-wise culture positivity of specimens

The number of positive cultures for WHO priority pathogens and other potentially pathogenic bacteria increased over the duration of hospitalization. On day 1 it was 58.7% (n=122, total n of cultures=208), 59.2% (n=109) on day 2 (total n of cultures=184), 64.3% (n=90) on day 3 (total n of cultures=140), 62.9% (n=83) on day 4 (total n of cultures=132), and 80% (n=80) on day 7 (total n of cultures=100) as shown in Table 2 and Figure 5.

**Table 2:** Day-wise culture positivity of specimens from all sites among study subjects

Day of hospitalization	No. of positive cultures	Total cultures	Positivity	Lower limit	Upper limit
1	122	208	58.7%	52.0%	65.3%
2	109	184	59.2%	52.1%	66.3%
3	90	140	64.3%	56.3%	72.2%
4	83	132	62.9%	54.6%	71.1%
7	80	100	80.0%	72.2%	87.8%

The number of positive cultures increased over time of hospitalization in all three categories. For the critical category, it was 6.7% (n=14) on day 1, 13.0% (n=24) on day 2, 20.0% (n=28) on day 3, 17.4% (n=23) on day 4, and 27.0% (n=27) on day 7 (p<0.05). For the high-priority category, it was 2.9% (n=6) on day 1, 2.2% (n=4) on day 2, 2.1% (n=3) on day 3, 1.5% (n=2) on day 4, and 4.0% (n=4) on day 7. The increase was not significant in the high WHO priority pathogens category. For the other pathogenic categories, it was 51.4% (n=107) on day 1, 52.2% (n=96) on day 2, 53.6% (n=75) on day 3, 55.3% (n=73) on day 4, and 69.0% (n=69) on day 7 (p<0.05) (Table 3).

**Table 3:** Day-wise distribution of microorganism categories and significant comparisons

WHO priority pathogen		Day of hospitalization										Significant comparisons
		1		2		3		4		7		
		n	%	n	%	n	%	n	%	n	%	
Critical	Yes	14	6.7	24	13.0	28	20.0	23	17.4	27	27.0	1 vs 3,4, 7, and 2 vs 7
High	Yes	6	2.9	4	2.2	3	2.1	2	1.5	4	4.0	
Pathogenic	Yes	107	51.4	96	52.2	75	53.6	73	55.3	69	69.0	1 vs 7
Total no. of cultures		208		184		140		132		100		

Table 4 shows the critical WHO pathogens that were most prevalent in the burns and plastic surgery ICU at 30.9% (n=47), compared to 13.5% (n=41) in the medical ICU and 9.1% (n=28) in the surgical ICU (p<0.001). Potentially pathogenic organisms were also highest in the burns and plastic surgery ICU at 73.7% (n=112) versus 48.4% (n=47) in the medical ICU and 52.3% (n=161) in the surgical ICU (p<0.001). Biological specimens (n=15) were collected from 11 patients across the different ICUs for nosocomial infection analysis. All specimens tested positive for WHO priority pathogens or other potentially pathogenic bacteria, with a total of 22 microorganisms isolated (17 critical WHO pathogens and five other potentially pathogenic bacteria), as shown in Tables 5-6. 

**Table 4:** ICU-wise distribution of pathogen groups

Pathogen Group	Burns and plastic surgery ICU (n=152)		Medical ICU (n=304)		Surgical ICU (n=308)		Total n=764		p-value
	N	%	n	%	n	%	n	%	
Critical	47	30.90	41	13.50	28	9.10	116	15.20	<0.001
High	2	1.30	7	2.30	10	3.20	19	2.50	0.441
Potentially pathogenic	112	73.70	147	48.40	161	52.30	420	55.00	<0.001

**Table 5:** Association between presence of WHO critical priority pathogen from the four sites and from the nosocomial specimen

WHO critical priority pathogen isolates from any of the sites	WHO Critical priority isolate from nosocomial specimen		Total
	Present	Absent	
Present	9 (90%)	1 (10%)	10
Absent	1 (10%)	0 (0%)	1
Total	10	1	11

**Table 6:** Association between the presence of other potentially pathogenic bacteria from the four sites and the nosocomial specimen

Other potentially pathogenic bacteria isolated from the four sites	Other potentially pathogenic isolates from the nosocomial specimen		Total
	Present	Absent	
Present	5 (45.5%)	6 (54.5%)	11
Absent	0 (0%)	0 (0%)	0
Total	5	6	11

## Discussion

The primary motivation for conducting this study at a tertiary care teaching hospital was the high patient load in the ICU and to study the prevalence and pattern of multidrug-resistant pathogens. Among the four selected sites, regardless of the day, the highest diversity of pathogenic flora was observed at site A, likely due to frequent contact with hospital surfaces (bed sheets, bed rails, etc.) and being commonly handled by attendants and staff. In the present study, gram-negative bacteria were the most frequently isolated pathogens, predominantly from the Enterobacteriaceae family, accounting for 43.2% of total isolates. Of these, 9.5% were ESBL-producing CRP-R Enterobacteriaceae (WHO critical priority pathogen, group-1), 16.5% were non-ESBL CRP-R, and 14.5% were non-ESBL CRP-S. Only 2.8% were ESBL-producing CRP-S. A study by Nseir et al. [4] reported a lower prevalence of ESBL-producing Enterobacteriaceae (9%) and a higher proportion of *P. aeruginosa* (16%), which may reflect differences in the flora of these healthcare settings.

Gram-positive bacteria in the present study accounted for 41.8% of total pathogens, with *S. aureus *(40.5%) as the most common. Of these, 30% were MRSA, 8.2% MSSA, and 2.3% VRSA. Although gram-negative bacteria were more prevalent overall, MRSA was the most frequently isolated individual pathogen across all sites, particularly at site D (36.1% of site D pathogens and 9.4% of total pathogens). A study by Gen et al. [9] on nasal carriage of *S. aureus* in acromegalic patients reported a lower carriage rate (13.3%) compared to normal individuals (43.4%). Another study by Abroo et al. [10] found a 19.6% nasal carriage of *S. aureus* in healthy medical and non-medical students, with 2.6% being MRSA and none being V-R. The difference in findings suggests that resistant flora, particularly MRSA, is acquired in the hospital setting, with colonization increasing over time.

The WHO critical priority pathogens were most frequently isolated from site D in the present study, comprising 5.8% of pathogens across all sites and 22.5% of site D isolates. These pathogens included ESBL-producing CRP-R Enterobacteriaceae (9.5%), CRP-R *P. aeruginosa* (6.5%), and CRP-R *A. baumannii* (2.9%). Other potentially pathogenic bacteria were more common at site A, accounting for 22% of total pathogens and 79.4% of site A isolates. Both *P. aeruginosa* and *A. baumanni*i represented 9.4% and 5.5% of isolates, respectively, with significant proportions being CRP-R (68.9% and 52.8%, respectively). In a study by Kateete et al. [11], 869 specimens were analyzed, with *P. aeruginosa* isolated in 5% and *A. baumannii* in 3% of cases. Of these, 24% of *P. aeruginosa* and 31% of *A. baumannii* were CRP-R. The higher incidence of CRP-R *P. aeruginosa* and *A. baumannii* in our setting may be attributed to the high patient load, limited human resources, and poor patient health literacy status, among other reasons.

In the present study, colonization rates increased with prolonged hospitalization across all sites. On day 1, overall colonization was 51.1%, with 6.7% attributable to WHO critical pathogens and 51% to other potentially pathogenic bacteria. By day 7, colonization rose to 86.1%, with critical WHO priority pathogens accounting for 27% and other potentially pathogenic bacteria for 69%. Sahin et al. [12] similarly reported an increase in colonization over time (51.1% to 86.1% in a week) in ICU patients (p<0.05). Of particular note, two patients who regularly washed their hands during prayers had sterile swabs from sites A and B for two consecutive days, underscoring the importance of hygiene in reducing nosocomial infection rates. Differences in flora were observed across ICUs in the present study. In the burn and plastic surgery ICU, *P. aeruginosa* (30.2%) was significantly more prevalent, with 76% being CRP-R. *Staphylococcus aureus* was the second most common pathogen (28.2%), with 88.3% being vancomycin-sensitive MRSA. These findings highlight the association between overcrowding, poor sanitation, and nosocomial infections. In the surgery ICU, *S. aureus* (41.8%) predominated, with 73.2% being vancomycin-sensitive MRSA, while in the medical ICU, *A. baumannii *(6.6%) was more common, equally divided between CRP-R and CRP-S strains.

Enterobacteriaceae were found across all ICUs. In a study by Latifi et al. [13] on burn patients, *S. aureus* was the most commonly isolated pathogen (55.1%), followed by *P. aeruginosa* (14.29%), Enterococcus (12.24%), and *Escherichia coli* (4%). They found *P. aeruginosa* to be the most frequently disseminated bacteria in burn wound infections. Similarly, Benchamkha et al. [14] reported *Staphylococcus* (37.7%), *P. aeruginosa* (19.8%), *Enterococcus faecalis*, and *Proteus mirabilis* (18.5%) as the dominant organisms in burn patients, with 22% of Staphylococci being methicillin-resistant and 66% of *Pseudomonas* and *Acinetobacter* showing multi-resistance. This difference is probably due to the difference in microbiological flora in these countries.

In the present study, the prevalence of WHO critical pathogens was highest in the burn and plastic surgery ICU (30.9%), followed by the medical (13.5%) and surgical (9.1%) ICUs (p<0.001). Similarly, potentially pathogenic organisms were most prevalent in the burn and plastic surgery ICU (73.7%), compared to the medical (48.4%) and surgical (52.3%) ICUs (p<0.001). This emphasizes the need for rigorous infection control measures and staff training to mitigate nosocomial infections. Colonization rates of WHO critical pathogens and other potentially pathogenic bacteria, in the present study, significantly increased with hospitalization duration across all ICUs, though this trend was not observed for WHO high-priority pathogens.

Finally, the Sepsis-related Organ Failure Assessment (SOFA) score [15] of the study subjects at the time of admission indicated that most were in critical condition upon entering the ICU. Several patients developed fever during follow-up, though many were lost due to LAMA or died before samples could be collected for nosocomial infection investigations. The high prevalence of nosocomial infections and associated mortality could be attributed to the absence of stringent protocols for infection screening and prevention in this setting.

Researchers suggest that comprehensive infection prevention and strategies outlined by individual healthcare practices and institutional initiatives emphasizing hand hygiene, environmental measures, leadership, personal protective equipment, consistent evidence-based practice, antimicrobial resistance campaigns, respiratory or cough etiquette, and evaluation can minimize HAIs [16]. Evidence from a systematic review indicates that providing readily accessible antiseptic hand rub dispensers significantly improves hand hygiene compliance among healthcare workers in acute care settings. Additionally, meta-analyses demonstrate that single-patient rooms help reduce the risk of colonization or infection by multidrug-resistant and other pathogens. In shared patient rooms, it is essential to consider the transmission routes of potential pathogens and maintain adequate spacing between beds to prevent droplet spread and ensure safe movement of staff and equipment. These findings highlight that hospital ward design plays a crucial role in effective infection prevention and control [17]. These factors formed a major contributor to the overloaded government setup under study.

The limitations of this study include its relatively small sample size, which may restrict the generalizability of the findings. Additionally, the short follow-up period of one week may not fully capture long-term trends in pathogen colonization or the development of nosocomial infections. The study's statistical power was limited by the sample size, making it difficult to establish significance for some associations, such as HAIs. Furthermore, as a single-center study, the results may not be representative of other healthcare settings, particularly those with different infection control protocols. Lastly, variability in patient comorbidities, treatment regimens, and ICU environments could have influenced colonization rates, introducing potential confounding factors into the study's outcomes. Hence, further longitudinal surveillance or multi-center studies with larger sample sizes can further validate these findings.

## Conclusions

This study highlights the significant burden of multidrug-resistant pathogens, particularly WHO critical priority pathogens, in ICU settings at a tertiary care hospital. The colonization of skin flora by pathogenic bacteria increased with hospitalization duration, with vancomycin-sensitive MRSA and CRP-R P. aeruginosa and Enterobacteriaceae being the most prevalent. Notably, the highest microbial load was observed in the burn and plastic surgery ICU, where infection control challenges were compounded by limited resources and overcrowding. The findings underscore the need for stringent infection control practices and regular monitoring of patient flora to prevent nosocomial infections. Proper hygiene, periodic disinfection, and improved staff training can significantly reduce the spread of HAIs.

## Figures and Tables

**Figure 6:**
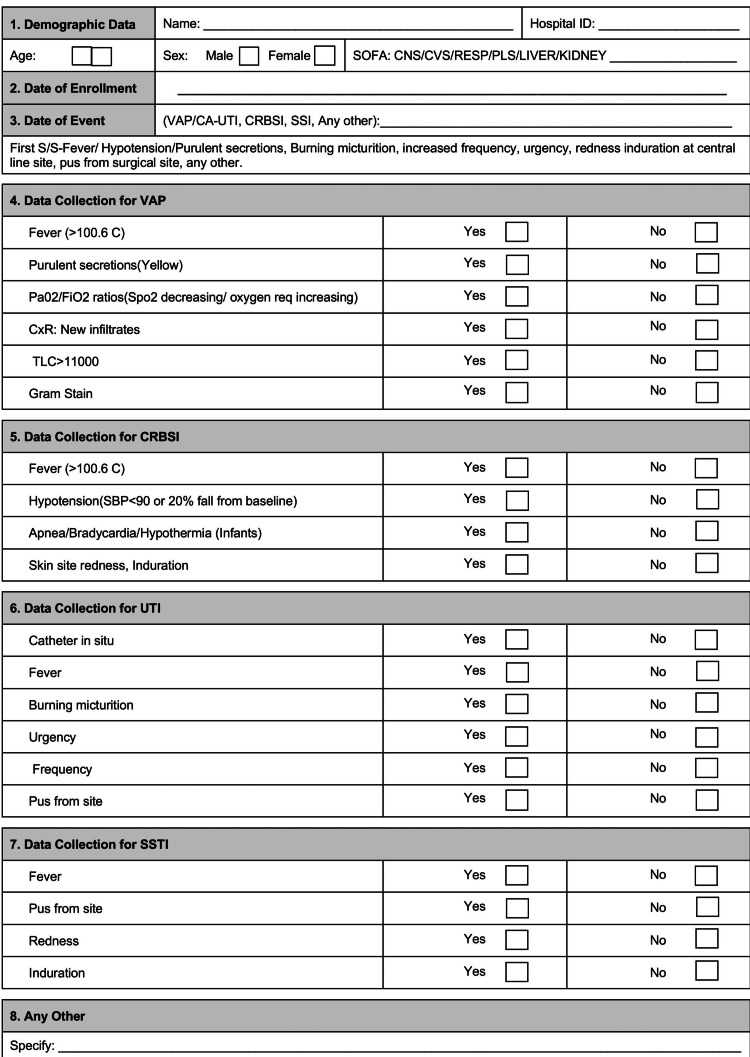
Data collection form

## Appendices

Appendix A

Figure 6 features the data collection form used to gather data on nosocomial infections and the relevant clinical signs from 11 patients included in the study.
